# Supplementary material for: Understanding the role of psychological distance in preventing the spread of kauri dieback
Source: PLoS One. 2025 Oct 27;20(10):e0334977. doi: 10.1371/journal.pone.0334977 (PMC12558519; doi:10.1371/journal.pone.0334977)
Supplement: S1 Appendix — (DOCX) [file pone.0334977.s001.docx]

**Appendices**

**Appendix A**

| *Direct Oblimin (∆ = 0) Rotated Component Loadings and Cronbach’s α for Psychological Distance Dimensions* | | | | |
| --- | --- | --- | --- | --- |
| Dimension | | Item | Single Factor Model Item Loadings (Cronback’s *α* = .82) | |
| Geographic | Where I live is largely immune from the effects of Kauri Dieback. | | | .67 |
|  | Kauri Dieback is currently a significant problem in my home city or town. | | | .63 |
|  | Kauri Dieback is more likely to impact communities that are far away from my home city or town. | | | .69 |
|  | When I think about Kauri Dieback, I usually think of communities that are far away from where I live. | | | .69 |
| Temporal | The worst impacts of Kauri Dieback are likely to occur far in the future. | | | .47 |
|  | Kauri Dieback poses an immediate threat to our native forests right now. | | | .33 |
|  | The slow spread of Kauri Dieback means we can delay significant action for a few more years. | | | .53 |
|  | I doubt that I will personally experience the direct effects of Kauri Dieback in my lifetime. | | | .70 |
| Social | Kauri Dieback is likely to have a big impact on people like me. | | | .60 |
|  | The biggest impacts of Kauri Dieback will be felt by people other than me. | | | .53 |
|  | I don’t think Kauri Dieback will have much of an impact on people I know. | | | .73 |
|  | People I know will directly experience the negative effects of Kauri Dieback. | | | .60 |
| Uncertainty | Most scientists agree about the impacts of Kauri Dieback on New Zealand forests. | | | .06 |
|  | In my opinion, the science about Kauri Dieback and its effects is far from settled. | | | .23 |
|  | I am uncertain whether current strategies to manage Kauri Dieback are effective. | | | .08 |
|  | I am uncertain about what the effects of Kauri Dieback will be. | | | .46 |
| *Notes:* | Bold items were reverse coded. All items were derived from Jones et al., (2017) | | | |
